# Impact of Calcineurin Inhibitor-Based Immunosuppression Maintenance During the Dialysis Period After Kidney Transplant Failure on the Next Kidney Graft Outcome: A Retrospective Multicenter Study With Propensity Score Analysis

**DOI:** 10.3389/ti.2023.11775

**Published:** 2023-09-15

**Authors:** Juliette Noelle, Valentin Mayet, Céline Lambert, Lionel Couzi, Bertrand Chauveau, Antoine Thierry, Laure Ecotière, Dominique Bertrand, Charlotte Laurent, Richard Lemal, Clarisse Grèze, Marine Freist, Anne-Elisabeth Heng, Paul-Olivier Rouzaire, Cyril Garrouste

**Affiliations:** ^1^ Service de Néphrologie Centre hospitalo-universitaire Clermont-Ferrand, Clermont-Ferrand, France; ^2^ Unité de Biostatistiques, Direction de la recherche clinique et d’ innovation, Centre hospitalo-universitaire Clermont-Ferrand, Clermont-Ferrand, France; ^3^ Service de Néphrologie, Transplantation, Dialyse et Aphérèses, Centre hospitalo-universitaire Bordeaux, Bordeaux, France; ^4^ Service de Pathologie, Centre hospitalo-universitaire de Bordeaux, Bordeaux, France; ^5^ Service de Néphrologie-Hémodialyse-Transplantation Rénale, Centre hospitalo-universitaire Poitiers, Poitiers, France; ^6^ Service de Néphrologie, Centre hospitalier régional universitaire rouen, Rouen, France; ^7^ Service d’Histocompatibilité et Immunogénétique, Centre hospitalo-universitaire Clermont-Ferrand, Clermont-Ferrand, France; ^8^ Service de Néphrologie et Dialyse, Centre hospitalier Emile Roux, Le Puy-en-Velay, France; ^9^ Service de Néphrologie Centre hospitalo-universitaire Clermont-Ferrand, Université Clermont Auvergne, Clermont-Ferrand, France; ^10^ EA 7453 CHELTER, Clermont-Ferrand, France

**Keywords:** kidney retransplant, kidney transplant failure, calcineurin inhibitor maintenance, waiting list, immunosuppression

## Abstract

The impact of immunosuppressive therapy (IS) strategies after kidney transplant failure (KTF) on potential future new grafts is poorly established. We assessed the potential benefit of calcineurin inhibitor (CNI)-based IS maintenance throughout the dialysis period on the outcome of the second kidney transplant (KT). We identified 407 patients who underwent a second KT between January 2008 and December 2018 at four French KT centers. Inverse probability of treatment weighting was used to control for potential confounding. We included 205 patients with similar baseline characteristics at KTF: a total of 53 received at least CNIs on the retransplant day (G-CNI), and 152 did not receive any IS (G-STOP). On the retransplant date, G-STOP patients experienced a longer pretransplant dialysis time, were more often hyperimmunized, and underwent more expanded-criteria donor KTs than G-CNI patients. During the second KT follow-up period, rejection episodes were similar in both groups. The 10-year survival rates without death and dialysis were 98.7% and 59.5% in G-CNI and G-STOP patients, respectively. In the multivariable analysis, CNI-based IS maintenance was associated with better survival (hazard ratio: 0.08; 95% confidence interval: 0.01–0.58, *p* = 0.01). CNI-based IS maintenance throughout the dialysis period after KTF may improve retransplantation outcomes.

## Graphical Abstract

**Figure:**
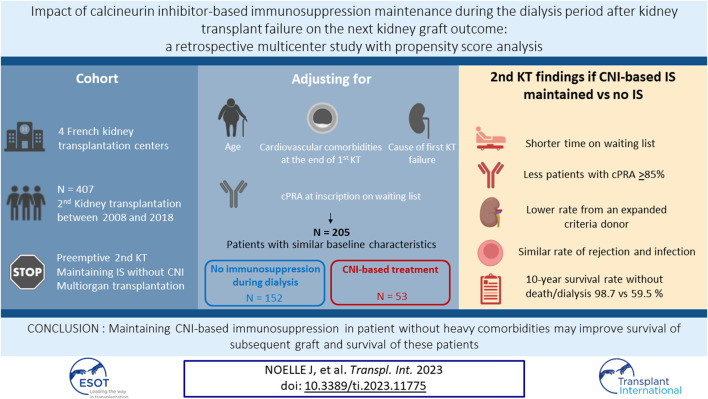

## Introduction

Since the 2000s, the number of patients waiting for a second transplant after kidney transplant failure (KTF) has increased year after year. Currently, they represent 13%–23% of patients on the waiting list ([1–4]) and approximately 14% of the transplantations performed in France [5]. The majority of these patients develop anti-human leucocyte antigen (HLA) antibodies after KTF, and immunosuppressive therapy (IS) is gradually withdrawn, thus limiting their access to a new transplant [6, 7]. They represent more than half of the hyperimmunized patients on the waiting list, defined by a calculated panel reactive antibody (cPRA) level ≥85% [1, 8, 9]. A prolonged wait time [1, 10] is associated with poorer survival of the second transplant [11–14] and increased mortality [11, 15, 16].

The optimal management of IS after KTF in potential candidates for a second kidney transplant (KT) remains uncertain [17]. Until recently, expert recommendations suggested a sequential decrease in IS with cessation of antimetabolites in the event of KTF, gradual withdrawal of calcineurin inhibitors (CNIs) with cessation between 1 month and 3 months, and a delayed cessation of steroids depending on residual diuresis and the occurrence of symptoms related to graft intolerance [18–20]. Recently, an American expert transplant group suggested stopping immunosuppressive drugs in the absence of transplantation 1 year after KTF [21]. IS withdrawal aims to minimize infectious, cardiovascular [22, 23], and neoplastic [24] risks in patients with KTF. On the other hand, the British Transplantation Society suggests maintenance of IS when a living donor transplant is planned in the year following KTF [25]. Indeed, recent studies have suggested a decrease in immunization that may allow better access to a subsequent KT if CNIs are maintained after KTF [26, 27], without an increased risk of cardiovascular or infectious events [28]. These divergences undoubtedly explain the very scarce literature on retransplant outcomes in patients with IS maintained throughout the dialysis period [29].

The objective of the present retrospective, multicenter, observational study was thus to evaluate the impact of CNI-based IS maintenance during the dialysis period until the new transplantation on the outcome of the second graft.

## Material and Methods

### Study Population

This retrospective, multicenter study was performed at four French adult KT centers (Clermont-Ferrand, Bordeaux, Rouen and Poitiers). Patients were selected using the Cristal prospective database. The inclusion criteria were patients over 18 years old who had undergone a second KT between 1 January 2008 and 31 December 2020 at the Clermont-Ferrand, Rouen, or Poitiers transplant centers or between 1 January 2016 and 31 December 2020 at the Bordeaux transplant center (because of a change of the computerized patient record systems). The exclusion criteria consisted of second preemptive transplantations, continuation of IS treatment without CNIs, and multiorgan transplantations.

### Data Collection

The following demographic, clinical, and biological data were collected: i) at the time of KTF—age, sex, body mass index, initial kidney disease, first transplant outcome and cause of allograft failure, PRA level, and the eventual presence of donor-specific anti-HLA antibodies (DSAs); ii) at the inscription on the waiting list for a second KT—PRA level and comorbidities (diabetes, stroke, ischemic heart disease, lower limb revascularization, neoplasia, and persistent post-KTF infection); iii) during the dialysis period—potential allograft nephrectomy, severe infection defined as an opportunistic infection [30] or requiring hospitalization [31], major cardiovascular events (hospitalization for acute coronary syndrome, cardiac arrhythmia, heart failure, lower limb revascularization, and stroke), and whether IS with CNIs was maintained; iv) at the retransplant initial hospitalization—induction therapy modalities, PRA level, eventual presence of DSAs (against the new KT), the type of donor (expanded criteria donor [32] or living donor), residual diuresis, and delayed graft function defined as the requirement of at least one dialysis session during the first week after transplantation [33]; and v) during the follow-up after the second KT—graft rejection episodes (Banff 2019 [34]), the appearance of DSAs, severe infection, major cardiovascular events, neoplasia, graft, and patient survival. Detection of anti-HLA antibodies was performed using the Luminex Single Antigen method (One Lambda, Canoga Park, CA) at the Clermont-Ferrand, Bordeaux, and Poitiers centers or Immucor Lifecodes (Immucor, Stamford, CT) at the Rouen center [35].

Oversight and study approval were provided by the Committee for Protection of Human Subjects (CPP SUD-EST VI) on 3 September 2019 (institutional review board 00008526) and by the National Consultative Committee on the Use of Health Research Information (14.510). No written consent was required for this study, but a non-opposition letter was sent to all patients in accordance with national legislation (MR-004 reference methodology) [36].

### Definition of Groups

Two groups of patients were defined according to the modality of management of IS in the period between the two KTs: i) the CNI group (G-CNI), defined by the continuation of IS including CNIs either as monotherapy or in combination with other IS (i.e., steroids, mycophenolate mofetil, azathioprine, and mTOR pathway inhibitors) during the entire period between the two KTs, and ii) the stop group (G-STOP), defined by the cessation of all IS during the intertransplant period.

### Statistics

Statistical analyses were performed with Stata software (version 15; StataCorp, College Station, Texas, USA). All tests were two-sided with an alpha level set at 5%. Categorical variables were expressed as number of patients and associated percentages, and continuous variables as mean ± standard deviation or median [25th; 75th percentiles], according to their statistical distribution.

Demographic and first transplant characteristics were compared between G-STOP and G-CNI using usual statistical tests: chi-squared test or Fisher’s exact test for categorical variables and Student’s t-test or Mann-Whitney test for continuous variables.

To assess the relationship between the group (G-STOP and G-CNI) and the primary and secondary endpoints, a propensity score (PS) analysis was implemented using the inverse probability of treatment weighting (IPTW) method [37, 38]. The PS was derived from the probability that treatment with a CNI would be continued for a given patient (G-CNI) conditional on confounders. The IPTW method consists of creating a “pseudo sample” of treated (G-CNI) and untreated (G-STOP) patients, weighting each patient by the inverse probability of receiving the treatment he or she actually received as follows: 1/PS in the G-CNI and 1/(1-PS) in the G-STOP. In practice, the probability of continuing CNI therapy was modeled using multiple logistic regression, and the estimated probability was used as the PS. Variables were selected for the PS based on clinical relevance: age at the end of the first transplant, cardiovascular comorbidities at the end of the first transplant, cause of first transplant failure, and cPRA level at the inscription on the waiting list for retransplant. Patients with missing cPRA levels at the inscription date were excluded from the analysis, as were patients with diabetes or infections because they were all G-STOP patients. Balance between groups was measured by standardized mean differences, calculated before and after weighting, and expressed as absolute values. A value greater than 0.2 was considered a sign of imbalance.

The primary outcome was a composite of dialysis and death after the second KT, presented as survival free of dialysis and death. This outcome was expressed as censored data and was estimated with the Kaplan-Meier method, and the groups were compared by the log-rank statistic. Multivariable analyses were performed with a Cox model (with the center as a random effect) considering covariables in terms of their significant results in univariate analysis (*p* < 0.10) as well as their clinical relevance^14,32^. The results are expressed as hazard ratios (HRs) and 95% confidence intervals (CIs).

Secondary outcomes were compared in both groups by mixed models, considering the center as a random effect: linear mixed models were used for continuous outcomes and generalized linear mixed models with the logit link function were used for binary outcomes.

Finally, exposure-adjusted rates were calculated as the total number of event episodes (including recurrent events) over the total duration of follow-up and are expressed per 100 patient-years (p-y).

## Results

### Patient Characteristics at the First-Graft Failure

**FIGURE 1:**
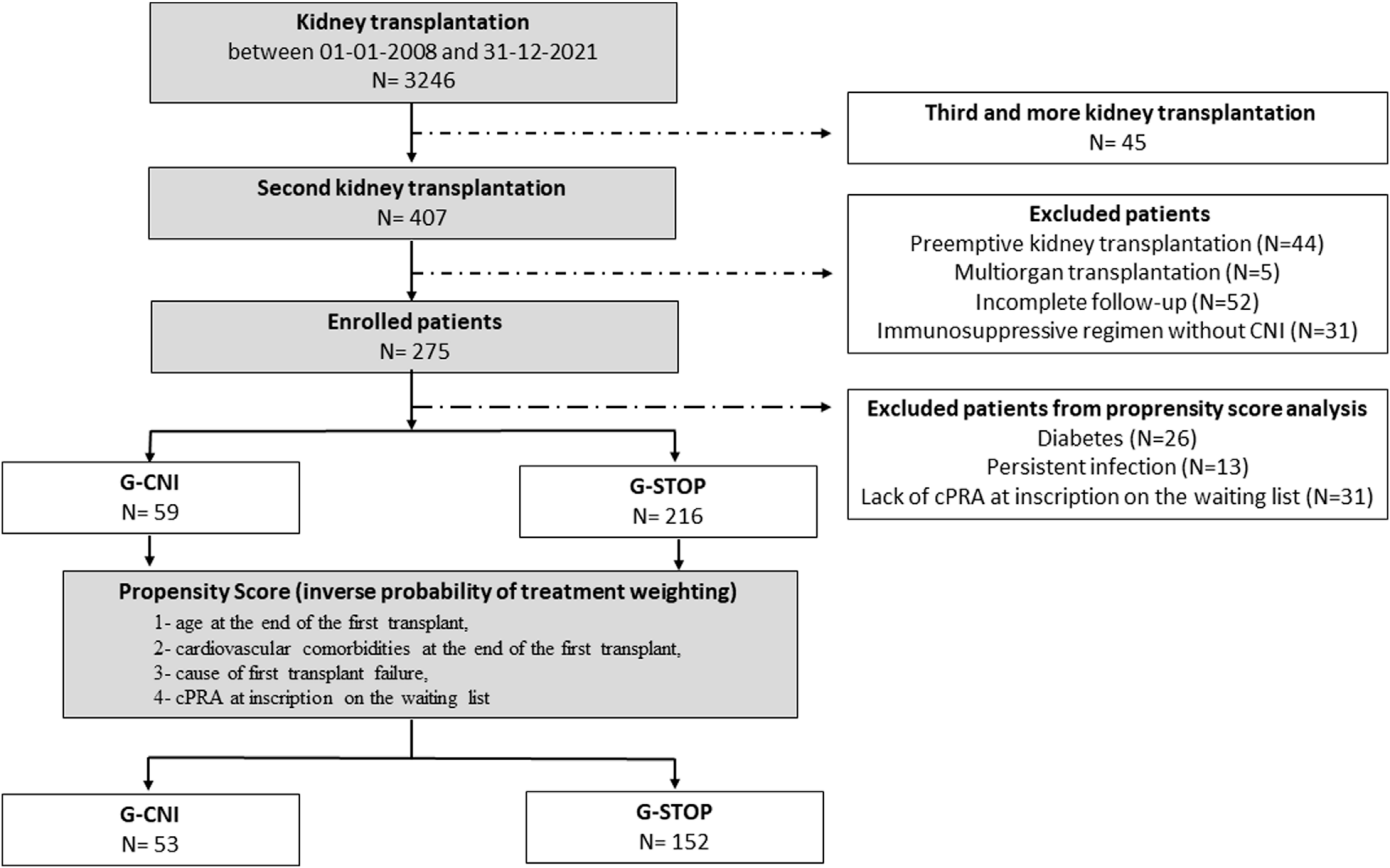
Flow chart of the study. CNI, calcineurin inhibitor; cPRA, calculated panel reactive antibody; G-CNI, group with immunosuppressive therapy maintenance; G-STOP, group with discontinued immunosuppressive therapy.

Among the 3246 KTs performed at the four centers during the study period, 407 patients (12.5%) received a second KT (Figure 1). Five patients with multiorgan transplantation were excluded, as well as 44 patients with preemptive KT, 31 patients with IS without CNIs, and 52 due to lack of data. A total of 275 patients were included, 216 in the G-STOP and 59 in the G-CNI. The median follow-up time after the second KT was 3.6 years [2.0; 7.0].

The characteristics of the patients before IPTW are depicted in Table 1. The 275 patients included were mainly men (64.7%), aged 49.7 ± 13.6 years at KTF. The primary cause of the first graft loss was rejection (61.8%). G-STOP patients compared to G-CNI patients had more diabetes at the end of the first KT (11.6% vs. 1.7%, *p* = 0.02), a shorter transplantation survival (92 months [34; 163] vs. 133 [87; 220], *p* = 0.003), and a higher cPRA at inscription on the waiting list for retransplant (51% [0; 86] vs. 5% [0; 70], *p* = 0.02). The rate of hyperimmunized patients (cPRA ≥85%) in the G-STOP and G-CNI was 26.7% and 13.2%, respectively (*p* = 0.04). Among G-CNI patients, 36 (61.0%) were treated with tacrolimus and 23 (39.0%) with cyclosporine. IS maintenance until the second KT consisted of CNI monotherapy in 19 patients (32.2%) and CNI combined with an antimetabolite or corticosteroid therapy in 30 patients (50.8%). Ten patients (17.0%) received triple IS.

**TABLE 1:** Characteristics at the first kidney transplant failure date of patients with (G-CNI) or without (G-STOP) calcineurin inhibitor maintenance throughout the intergraft period.

	Total (*n* = 275)	G-STOP (*n* = 216)	G-CNI (*n* = 59)	*p*
Age at the end of G1 (years)	49.7 ± 13.6	49.1 ± 13.6	52.0 ± 13.3	0.15
Male sex	178 (64.7)	140 (64.8)	38 (64.4)	0.95
Body mass index (kg/m^2^)	24.4 ± 4.6	24.6 ± 4.7	23.7 ± 3.9	0.13
Causal nephropathy				
Vascular nephropathy	14 (5.1)	13 (6.0)	1 (1.7)	0.14
Genetic nephropathy	57 (20.7)	40 (18.5)	17 (28.8)	
Glomerulonephritis	126 (45.8)	96 (44.4)	30 (50.8)	
Diabetic nephropathy	6 (2.2)	6 (2.8)	0 (0.0)	
Urological	44 (16.0)	39 (18.1)	5 (8.5)	
Other	28 (10.2)	22 (10.2)	6 (10.2)	
Comorbidities at the end of G1				
Diabetes	26 (9.5)	25 (11.6)	1 (1.7)	0.02
Cardiovascular disease^a^	37 (13.5)	28 (13.0)	9 (15.3)	0.65
Infections^b^	13 (4.7)	13 (6.0)	0 (0.0)	0.08
Solid cancer	25 (9.1)	18 (8.3)	7 (11.9)	0.40
Recurrent skin cancer	6 (2.2)	3 (1.4)	3 (5.1)	0.12
Hemopathy	5 (1.8)	4 (1.9)	1 (1.7)	1.00
G1 duration (months)	106 [43; 176]	92 [34; 163]	133 [87; 220]	0.003
Cause of G1 failure				
Rejection	170 (61.8)	132 (61.1)	38 (64.4)	0.11
Infection	10 (3.6)	10 (4.6)	0 (0.0)	
IFTA	28 (10.2)	20 (9.3)	8 (13.6)	
Vascular	37 (13.5)	33 (15.3)	4 (6.8)	
Causal nephropathy recurrence	30 (10.9)	21 (9.7)	9 (15.2)	
Presence of DSAs at the end of G1	62/228 (27.2)	46/173 (26.6)	16/55 (29.1)	0.72
cPRA at graft failure (%) (*n =* 244)	48 [0; 83]	51 [0; 86]	5 [0; 70]	0.02
cPRA at graft failure ≥85%	58/244 (23.8)	51/191 (26.7)	7/53 (13.2)	0.04

Data are expressed as the number of patients (associated percentage), mean ± standard deviation or median [25th; 75th percentiles]. cPRA, calculated panel reactive antibody; DSA, donor-specific antibody; G1, first graft; G-CNI, group with immunosuppressive therapy maintenance; G-STOP, group with discontinued immunosuppressive therapy; IFTA, interstitial fibrosis and tubular atrophy.

^a^ Cardiovascular comorbidities: cerebrovascular accident, ischemic heart disease and/or obliterating arteriopathy of the lower limbs (surgical treatment).

^b^ Infections: numerous or persistent at the time of kidney transplant failure.

### Patient Characteristics at the First-Graft Failure After IPTW

The characteristics at the time of KTF of the 205 patients included in the PS analysis are summarized in Table 2. After applying the IPTW method, the G-STOP and G-CNI were well balanced (standardized mean differences <20%) for the variables included in the IPTW model: age, cardiovascular comorbidities, the cause of first-transplant failure, and the cPRA level at inscription on the waiting list for retransplant.

**TABLE 2:** Characteristics at the first kidney transplant failure date of patients with (G-CNI) or without (G-STOP) calcineurin inhibitor maintenance throughout the intergraft period before and after applying inverse probability weighting.

	Before IPTW			After IPTW		
	G-STOP (*n* = 152)	G-CNI (*n* = 53)	SMD	G-STOP	G-CNI	SMD
Age at the end of G1 (years)	47.9 ± 13.8	51.9 ± 13.2	0.29	48.9 ± 14.0	48.4 ± 12.8	0.04
Male sex	97 (63.8)	35 (66.0)	0.05	(64.7)	(59.2)	0.11
Body mass index (kg/m^2^)	24.4 ± 4.7	23.7 ± 3.7	0.18	24.4 ± 4.7	23.5 ± 3.6	0.22
Causal nephropathy						
Vascular nephropathy	11 (7.2)	1 (1.9)	0.26	(8.1)	(1.3)	0.32
Genetic nephropathy	25 (16.5)	15 (28.3)	0.29	(16.1)	(36.3)	0.47
Glomerulonephritis	73 (48.0)	26 (49.1)	0.02	(48.2)	(44.3)	0.08
Diabetic nephropathy	0 (0.0)	0 (0.0)	NA	(0.0)	(0.0)	NA
Urological	26 (17.1)	5 (9.4)	0.23	(16.4)	(8.0)	0.26
Other	17 (11.2)	6 (11.3)	0.00	(11.2)	(10.1)	0.04
Comorbidities at the end of G1						
Diabetes	0 (0.0)	0 (0.0)	NA	(0.0)	(0.0)	NA
Cardiovascular disease^a^	18 (11.8)	8 (15.1)	0.10	(12.7)	(11.6)	0.03
Infections^b^	0 (0.0)	0 (0.0)	NA	(0.0)	(0.0)	NA
Solid cancer	13 (8.6)	7 (13.2)	0.15	(9.8)	(10.4)	0.02
Recurrent skin cancer	1 (0.7)	3 (5.7)	0.29	(0.7)	(4.1)	0.22
Hemopathy	3 (2.0)	1 (1.9)	0.01	(1.8)	(1.4)	0.03
G1 duration (months)	92 [36; 167]	133 [90; 217]	0.44	104 [43; 172]	120 [44; 205]	0.16
Cause of G1 failure						
Rejection	96 (63.2)	34 (64.1)	0.02	(63.5)	(59.5)	0.08
Infection	0 (0.0)	0 (0.0)	NA	(0.0)	(0.0)	NA
IFTA	14 (9.2)	6 (11.3)	0.07	(9.6)	(7.7)	0.07
Vascular	23 (15.1)	4 (7.6)	0.24	(13.4)	(20.1)	0.18
Causal nephropathy recurrence	19 (12.5)	9 (17.0)	0.13	(13.5)	(12.7)	0.02
Presence of DSAs at the end of G1	30/120 (25.0)	13/49 (26.5)	0.04	(22.9)	(29.7)	0.15
cPRA at graft failure (%)	50 [0; 84]	5 [0; 70]	0.32	44 [0; 83]	56 [0; 83]	0.06
cPRA at graft failure ≥85%	37 (24.3)	7 (13.2)	0.29	(22.2)	(23.8)	0.04

Data are expressed as the number of patients (associated percentage), mean ± standard deviation, or median [25th; 75th percentiles]. cPRA, calculated panel reactive antibody; DSA, donor-specific antibody; G1, first graft; G-CNI, group with immunosuppressive therapy maintenance; G-STOP, group with discontinued immunosuppressive therapy; IFTA, interstitial fibrosis and tubular atrophy; IPTW, inverse probability of treatment weighting; NA, not applicable; SMD, standardized mean difference.

^a^ Cardiovascular comorbidities: cerebrovascular accident, ischemic heart disease, and/or obliterating arteriopathy of the lower limbs (surgical treatment).

^b^ Infections: numerous or persistent at the time of kidney transplant failure.

### Waiting Time and Characteristics of the Patients After the Second KT After IPTW

The median time on dialysis until the second KT was 21 months [11; 43]. This value was significantly lower in the G-CNI than in the G-STOP (16 [5; 26] vs. 37 [22; 64], respectively, *p* < 0.001). The waiting times from relisting to the second KT were 16 months [8; 23] in the G-CNI and 27 months [13; 48] in the G-STOP (*p* = 0.06) (Table 3).

**TABLE 3:** Intergraft period and second transplantation outcomes after inverse probability weighting.

	Total	G-STOP	G-CNI	*p*
Intergraft period				
Pretransplant dialysis time (months)	21 [11; 43]	37 [22; 64]	16 [5; 26]	<0.001
Time on the waiting list (months)	19 [9; 37]	27 [13; 48]	16 [8; 23]	0.06
Transplantectomy and causes	(24.5)	(34.1)	(15.4)	0.06
Thrombosis	(39.5)	(34.6)	(48.3)	0.94
Graft intolerance syndrome	(52.2)	(52.5)	(51.7)	
Infection	(1.4)	(2.2)	(0.0)	
Surgical reason	(1.2)	(1.8)	(0.0)	
Other	(5.7)	(8.9)	(0.0)	
Second transplantation				
cPRA at D0 (%)	76 [25; 93]	87 [55; 96]	67 [0; 84]	0.001
cPRA at D0 ≥ 85%	(39.4)	(55.2)	(23.9)	<0.001
Anti-HLA antibodies at D0	(82.0)	(91.5)	(72.9)	0.047
Presence of DSAs at D0	(16.5)	(16.7)	(16.4)	0.99
HLA-A/B/DR antigen mismatches (0–6)	4 [2; 4]	3 [2; 4]	4 [2; 4]	0.92
HLA- DR antigen mismatches, N = 2	(14.5)	(16.9)	(12.3)	0.39
Cold ischemia time (minutes)	940 [688; 1,110]	960 [742; 1,208]	935 [620; 1,051]	0.03
Living donor	(10.6)	(7.8)	(13.3)	0.26
Expanded criteria donor	(36.4)	(43.2)	(29.9)	0.01
Residual urine output ≥500 mL	(29.1)	(13.8)	(44.1)	0.06
Induction treatment				
No induction treatment	(0.5)	(1.0)	(0.0)	0.27
Thymoglobulin	(78.8)	(84.0)	(73.8)	
Basiliximab	(20.7)	(15.0)	(26.2)	
Delayed graft function	(17.7)	(25.8)	(9.9)	0.001
Evolution after second transplantation				
Rejection	(18.7)	(22.1)	(15.5)	0.47
Humoral	(14.0)	(14.6)	(13.4)	0.95
Cellular	(5.4)	(8.8)	(2.1)	<0.001
Development of DSA	(9.7)	(10.4)	(8.9)	0.52
Return to dialysis and causes	(9.6)	(18.3)	(1.3)	0.004
Rejection	(45.1)	(48.4)	(0.0)	NA
Infection	(2.9)	(3.1)	(0.0)	
IFTA	(27.9)	(29.9)	(0.0)	
Vascular	(24.1)	(18.6)	(100.0)	
Causal nephropathy recurrence	(0.0)	(0.0)	(0.0)	
Death and causes	(4.7)	(9.7)	(0.0)	<0.001
Infection	(19.4)	(19.4)	—	NA
Cancer	(7.8)	(7.8)	—	
Cardiovascular	(29.9)	(29.9)	—	
Other	(42.9)	(42.9)	—	

Data are expressed as the number of patients (percentage) or median [25th; 75th percentiles]. cPRA, calculated panel reactive antibody; D0, day of transplantation; DSA, donor-specific antibody; G-CNI, group with immunosuppressive therapy maintenance; G-STOP, group with discontinued immunosuppressive therapy; HLA, human leucocyte antigen; IFTA, interstitial fibrosis and tubular atrophy; NA, not applicable.

G-CNI patients, compared to G-STOP patients, had a lower median cPRA level at the time of the second KT (67% [0; 84] vs. 87% [55; 96], *p* = 0.001). The rate of hyperimmunized patients was also lower in the G-CNI: 23.9% versus 55.2% in the G-STOP (*p* < 0.001). The numbers of patients transplanted with preformed DSAs and induction treatment were comparable between the groups (Table 3).

Patients in the G-STOP were more frequently transplanted with an expanded criteria donor graft (43.2% vs. 29.9% in the G-CNI, *p* = 0.01). Hyperimmunized patients, compared with patients with cPRA levels <85%, were more likely to receive a kidney transplant from an expanded criteria donor [44.1% and 32%, respectively (*p* = 0.005)]. On the day of the second KT, 44.1% of G-CNI patients had a residual diuresis ≥500 mL compared to 13.8% in G-STOP (*p* = 0.06). The delayed graft function rate was 9.9% in the G-CNI and 25.8% in the G-STOP (*p* = 0.001). The numbers of patients transplanted with preformed DSA and an induction treatment were comparable between the groups (Table 3). Data before IPTW are presented in Supplementary Table SI.

### Outcome After the Second KT After IPTW

**FIGURE 2:**
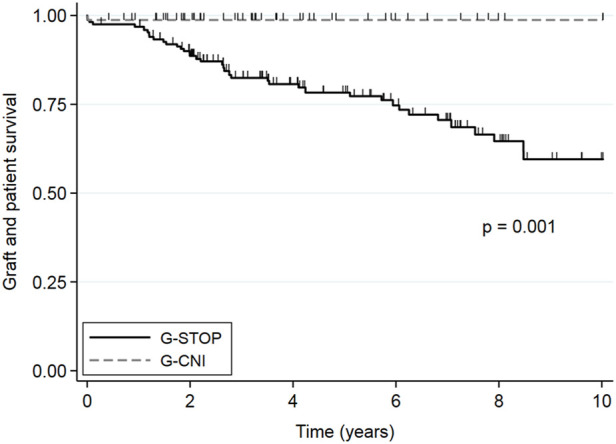
Overall second kidney transplant survival (without death or kidney transplant failure) in patients with immunosuppressive therapy maintenance until the second graft (G-CNI) or discontinued therapy (G-STOP). Data are presented after the inverse probability of treatment weighting.

After the second KT, 10 years survival free of dialysis and death was significantly better in the G-CNI than in the G-STOP (HR: 0.06, 95% CI: 0.01–0.30, *p* = 0.001) (Figure 2), with 10 years survival rates of 98.7% and 59.5%, respectively. In multivariable analysis after adjustment for expanded criteria donor, rejection, delayed graft function, age at second KT, graft survival time from the primary transplant, and rejection as etiology of first graft failure, continuation of CNIs between the two KTs was associated with a better 10 years survival free of dialysis and death (HR: 0.08, 95% CI: 0.01–0.58, *p* = 0.01) (Table 4). The difference in survival also remained significant after sensitivity analysis excluding second living donor transplants, with a 10 years survival rate of 98.5% in the G-CNI versus 56.4% in the G-STOP (HR: 0.06, 95% CI: 0.01–0.30, *p* = 0.001). Data on survival before IPTW are presented in Supplementary Figure S1.

**TABLE 4:** Multivariable analysis of the factors associated with 10 years survival free of dialysis and death in patients after second renal transplantation after inverse probability weighting.

	HR	95% CI	*p*
Second transplant			
CNI maintenance (vs. stop)	12.50	1.72; 100.0	0.01
Expanded criteria donor	0.40	0.09; 1.79	0.23
Recipient age (years)	0.97	0.94; 1.01	0.06
Rejection	0.32	0.20; 0.52	<0.001
Cold ischemia time (minutes)	1.00	1.00; 1.01	0.77
First transplant			
Graft survival (years)	1.01	1.01; 1.01	0.002
Rejection	0.42	0.27; 0.67	<0.001

CI, confidence interval; CNI, calcineurin inhibitor; HR, hazard ratio.

A return to dialysis was observed in 18.3% of G-STOP patients compared to 1.3% of G-CNI patients (*p* = 0.004). The main cause of graft loss was rejection (45.1%). The number of humoral rejections and the occurrence of DSA were comparable in the two groups, but there was less cellular rejection in the G-CNI than in the G-STOP (2.1% vs. 8.8%, respectively, *p* < 0.001). All deaths were observed in the G-STOP (Table 3; Supplementary Table SI).

### Major Cardiovascular, Infectious, and Neoplastic Events

**FIGURE 3:**
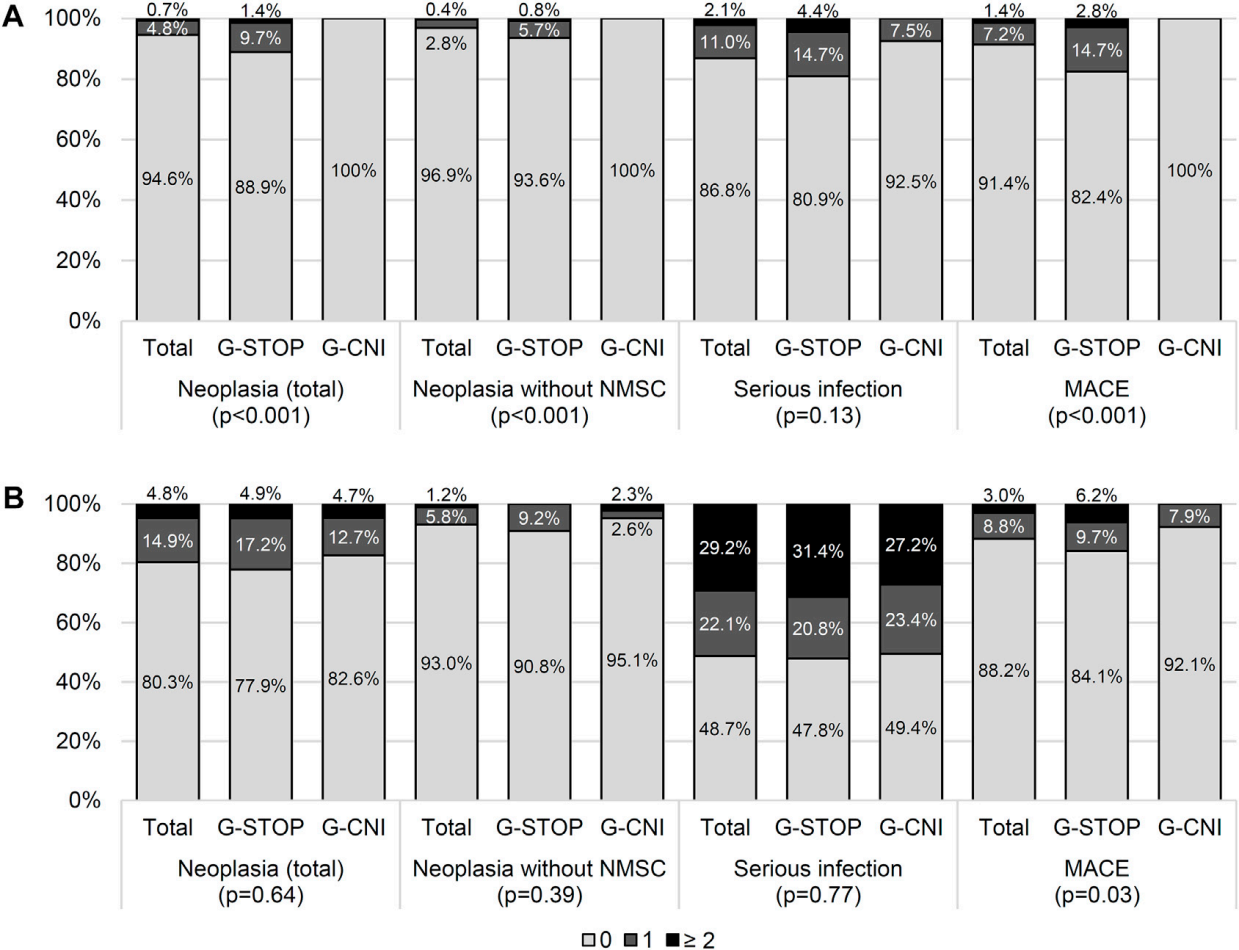
Adverse events between the two kidney transplants **(A)** and after the second transplant **(B)** in patients with immunosuppressive therapy maintenance (G-CNI) or discontinued therapy (G-STOP). Data are presented after the inverse probability of treatment weighting. MACE, major cardiovascular event; NMSC, non-melanoma skin cancer.

In the period between the two KTs, the serious infectious event rates and their exposure-adjusted rates (patient-years) in the G-CNI and G-STOP were similar (Figure 3A; Supplementary Figure S2). The rates of cardiovascular events and neoplasia and their exposure-adjusted rates were significantly lower in the G-CNI than in the G-STOP (Figure 3A; Supplementary Figure S2).

At the last follow-up after the second KT, the rates of patients with neoplastic events were similar in the G-CNI and G-STOP (Figure 3B). The rate of cardiovascular events was lower in the G-CNI than in the G-STOP (7.9% and 15.9%, respectively, *p* = 0.04) (Figure 3B). The serious infectious event rates were similar in the G-CNI and G-STOP (Figure 3B), but the exposure-adjusted rate was higher in the G-CNI than in the G-STOP (28.2/100 p-y and 22.8/100 p-y, respectively; *p* = 0.02) (Supplementary Figure S2).

Overall, after the first KTF, patients in the G-CNI and the G-STOP had a higher exposure-adjusted rate of serious infection (22.0/100 p-y and 15.3/100 p-y, respectively; *p* < 0.001) but a lower rate of major cardiovascular events (1.5/100 p-y and 4.8/100 p-y, respectively; *p* < 0.001). The exposure-adjusted rate of neoplasia was similar in both groups (Supplementary Figure S2).

## Discussion

To our knowledge, this retrospective multicenter study is the first report relative to the impact of maintaining IS with CNIs in patients with KTF throughout the dialysis period on the second KT. Our results show that the maintenance of CNI-based IS therapy during the dialysis period is associated with a lower HLA immunization rate, lower waiting time before retransplantation, and less use of expanded criteria donors. Remarkably, G-CNI patients had a better survival free of dialysis and death at 10 years than G-STOP patients.

In the literature, the negative impact of the dialysis waiting time after KTF on the subsequent KT outcome and increased mortality is well documented [11, 14, 21]. In a recent study of 911 patients from the ANZDATA registry, each year spent on dialysis after KTF was associated with a 5% increase in the risk of death (mainly from cardiovascular or infectious events) as well as a greater risk of acute rejection and graft failure after the second KT [11]. The impact of the second KT on survival seems to be particularly beneficial when it takes place in the first 3 years after the return to dialysis [15]. One way to explain the two-fold shorter dialysis wait time in the G-CNI compared to the G-STOP in our study is the lower immunization after KTF in the G-CNI before and after IPTW. Indeed, despite a similar cPRA level at re-registration after PS analysis, G-CNI patients had a significantly lower median cPRA level at the time of the second KT. Moreover, the rate of hyperimmunized subjects was also lower in the G-CNI than in the G-STOP. These results are consistent with those previously reported in the literature. Thus, in 77 Spanish patients who experienced KTF, the cessation of CNIs in the first 6 months was significantly associated with the development of DSA with respect to the first graft (odds ratio: 23.2, 95% CI: 5.3–100.6, *p* < 0.001) [27]. In another study performed in the USA in 119 patients with KTF, 68% of patients with discontinued IS were hyperimmunized after 24 months, compared to 8% of patients with IS continuation that included a CNI (*p* < 0.001) [26]. The latter had better access to retransplantation (46% vs. 29%) and a shorter median waiting time between relisting and the second KT (17 [7; 55] vs. 36 [3; 72] months) ^23^. The significantly higher rate of hyperimmunized patients in the G-STOP group may explain in part why these patients received more expanded criteria deceased donor allografts [32]. Indeed, French biomedical agency gives priority access to KT for patients with PRA levels >85%. In comparison with other donor types, the use of expanded criteria deceased donor kidneys for transplantation has a significant negative impact on graft survival [39–41] and death [40], whether it is a first transplant or a retransplant [14].

While the rate of humoral rejection was similar in both groups, we observed a lower rate of cellular rejection in the G-CNI than in the G-STOP after the second KT. The rates of second transplants with preformed DSAs and *de novo* DSAs were similar in the two groups, which may explain the similar humoral rejection rates in the two groups [13]. Healthy et al. previously reported risk factors for acute rejection after retransplant as a shorter primary graft survival, rejection in the first KTF, and a long time spent on dialysis [14]. We can hypothesize the role of alloreactive memory T-cells [42, 43] acquired during the first allograft period but also during the dialysis period [29]. Indeed, a recent German retrospective study [29] reported a significantly lower rate of T-cell-mediated rejection of the second KT and better graft survival (*p* = 0.02) in patients with *in situ* previous transplants who also usually had CNI maintenance compared to patients with first allograft nephrectomy who also usually discontinued therapy. The authors observed less T-cell alloreactivity measured by ELISPOT assay against the pretransplant donor in the group with CNI maintenance for a prolonged period compared to patients with discontinued treatment due to transplantectomy [29].

The benefit-risk balance of IS maintenance until a new KT is widely debated in the literature. Some retrospective cohort studies have observed higher rates of major cardiovascular, infectious, or tumor events in patients with IS maintenance [22, 23]. However, the IS regimens continued after KTF are highly variable and could include only low-dose corticosteroids. In our previous work, we reported an increased risk of infection associated with the continuation of corticosteroids but not with CNI maintenance therapy [44]. In the present work, we did not observe an increase in these adverse events before the second KT in the G-CNI. Our results are similar to the most recent data available [26]. In a study of 102 patients with KTF, mortality was similar in patients in whom IS was discontinued early within 3 months after KTF (*n* = 52) and in patients (*n* = 50) in whom IS was continued with antimetabolites and/or CNIs [45]. A Canadian prospective registry did not observe any difference in the infectious rate between patients in whom IS was continued after KTF and those in whom IS was discontinued [28]. However, we observed higher exposure-adjusted rates (p-y) of serious infectious events in G-CNI after the second KT. Future studies will have to be vigilant regarding this point.

The current work includes several limitations. First, due to the retrospective nature of the study, major differences between the two groups were observed, such as the rates of diabetes at the end of the first KT, persistent infections at the time of KTF, and PRAs level at relisting in the G-CNI. We thus proposed a PS analysis using the IPTW method to reduce the effect of these confounding factors that may have influenced survival. However, we cannot exclude the existence of factors not accounted for [46]. Indeed, there seem to be patient profiles in which IS is more likely to be maintained, such as the persistence of significant diuresis [47] or a living donor transplant [48]. Recently, a prospective Canadian study showed a similar profile of patients on IS therapy after KTF. Other underlying confounding factors are probably unknown, such as social level [49] and ease of access to care [50, 51]. One of the main decision-making factors remains the prescribing habits of transplant nephrologists, as highlighted by recent surveys in the USA [48, 52] and France [44]. Only a prospective randomized study will be able to overcome the confounding factors. Second, as this study focused on patients who had access to a second KT, we cannot exclude the possibility that patients who had continued CNI-based IS after KTF experienced serious adverse events with abandonment of the retransplant plan or even death without being counted. Additionally, we were not able to access the date of cessation of IS treatment and thus establish its temporality in relation to the possible occurrence of an adverse event. However, we previously carried out a preliminary retrospective study of 119 KT patients relisted after KTF at four French adult KT centers. We did not report an increased risk of infectious, neoplastic, or cardiovascular events or death in patients in whom a CNI was continued for more than 3 months after KTF [44]. Furthermore, in the present cohort, according to the records, only one patient who was not immunized had IS interruption due to infection 2 months before retransplant. He subsequently developed acute antibody-mediated rejection with preformed DSAs against the new transplant. Finally, we chose to consider the maintenance of IS treatments only if CNIs were maintained. Indeed, only CNIs were associated with lower immunization during the inter-transplant period [27, 44, 53]. For the cohorts reported in the literature [48], G-CNI patients received heterogeneous treatments, with only one-third of patients on CNIs alone and almost one-fifth of patients on triple IS. Furthermore, residual CNI levels are rarely measured in patients after KTF and therefore were not collected. Only a recent English study of 48 adult KTF transplant recipients reported a residual tacrolimus level ≥3 ng/ml as protective against the development of alloimmunization [54]. Further studies are necessary to determine the optimal CNI-based IS protocols after KTF.

Our study shows that after KTF, maintaining CNI-based IS in a cohort of patients without heavy comorbidities may reduce the risk of immunization, shorten the waiting time, and provide better access to standard criteria donor grafts. These strategies may improve the survival of the subsequent graft and these patients.

## Data Availability

The raw data supporting the conclusion of this article will be made available by the authors, without undue reservation.

## Supplementary Material

Supplementary Figure S1 Overall second kidney transplant survival (without death or kidney transplant failure) in patients with immunosuppressive therapy maintenance until the second graft (G-CNI) or discontinued therapy (G-STOP). Data are presented before the inverse probability of treatment weighting.

Supplementary Figure S2 Exposure-adjusted rates of serious complications in patients with immunosuppressive therapy maintenance until the second graft (G-CNI) or discontinued therapy (G-STOP). Data are presented after the inverse probability of treatment weighting. KT, kidney transplant; MACE, major cardiovascular event; NMSC, non-melanoma skin cancer. **p* < 0.05.

## Abbreviations

CI, confidence interval; CNI, calcineurin inhibitor; cPRA, calculated panel reactive antibody; DSA, donor-specific antibody; G-CNI, group with immunosuppressive therapy maintenance; G-STOP, group with discontinued immunosuppressive therapy; HLA, human leucocyte antigen; HR, hazard ratio; IPTW, inverse probability of treatment weighting; IS, immunosuppressive therapy; KT, kidney transplant; KTF, kidney transplant failure; PS, propensity score; p-y, patient-years.
