# Supplementary material for: Impact of Calcineurin Inhibitor-Based Immunosuppression Maintenance During the Dialysis Period After Kidney Transplant Failure on the Next Kidney Graft Outcome: A Retrospective Multicenter Study With Propensity Score Analysis
Source: Transpl Int. 2023 Sep 15;36:11775. doi: 10.3389/ti.2023.11775 (PMC10548547; doi:10.3389/ti.2023.11775)
Supplement: Supplementary file 1 [file Table1.DOCX]

Table S-I: Intergraft period and second transplantation outcomes before inverse probability weighting (n=275).

|  | **Total**  **(n=275)** | **G-STOP**  **(n=216)** | **G-CNI**  **(n=59)** | **p** |
| --- | --- | --- | --- | --- |
| **Intergraft period** |  |  |  |  |
| Pretransplant dialysis time (months) | 31 [17; 58] | 38 [22; 66] | 12 [5; 21] | **<0.001** |
| Time on waiting list (months) | 23 [11; 42] | 26 [14; 48] | 15 [7; 23] | **<0.001** |
| Transplantectomy and causes | 81 (29.5) | 76 (35.2) | 5 (8.5) | **<0.001** |
| Thrombosis | 25/72 (34.7) | 23/67 (34.3) | 2/5 (40.0) | 0.96 |
| Graft intolerance syndrom | 36/72 (50.0) | 34/67 (50.7) | 2/5 (40.0) |  |
| Infection | 2/72 (2.8) | 1/67 (1.5) | 1/67 (1.5) |  |
| Surgical reason | 2/72 (2.8) | 2/67 (3.0) | 0/5 (0.0) |  |
| Other | 7/72 (9.7) | 7/67 (10.5) | 0/5 (0.0) |  |
| **Second transplantation** |  |  |  |  |
| cPRA at D0 (%) | 86 [50; 96] | 89 [68; 97] | 53 [0; 84] | **<0.001** |
| cPRA at D0 ≥ 85% | 142/273 (52.0) | 128/215 (59.5) | 14/58 (24.1) | **<0.001** |
| Anti-HLA antibodies at D0 | 242/272 (89.0) | 200/214 (93.5) | 42/58 (72.4) | **<0.001** |
| Presence of DSA at D0 | 44 (16.0) | 37 (17.1) | 7 (11.9) | 0.33 |
| Cold ischemia time (minutes) | 940 [712; 1167] | 957 [744; 1205] | 892 [587; 1050] | **0.01** |
| Living donor | 25 (9.1) | 17 (7.9) | 8 (13.6) | 0.14 |
| Expanded criteria donor | 124 (45.1) | 100 (46.3) | 24 (40.7) | 0.44 |
| Residual urine output ≥ 500 mL | 58/248 (23.4) | 30/194 (15.5) | 28/54 (51.9) | **<0.001** |
| Induction treatment |  |  |  | **0.005** |
| No induction treatment | 1 (0.4) | 1 (0.5) | 0 (0.0) |  |
| Thymoglobulin | 232 (84.4) | 189 (87.5) | 43 (72.9) |  |
| Basiliximab | 42 (15.3) | 26 (12.0) | 16 (27.1) |  |
| Delayed graft function | 67 (24.4) | 61 (28.2) | 6 (10.2) | **0.006** |
| **Evolution after second transplantation** | |  |  |  |
| Rejection | 58 (21.1) | 50 (23.1) | 8 (13.6) | 0.11 |
| Humoral | 36 (13.1) | 30 (13.9) | 6 (10.2) | 0.46 |
| Cellular | 27 (9.8) | 24 (11.1) | 3 (5.1) | 0.14 |
| Development of DSA | 28 (10.2) | 22 (10.2) | 6 (10.2) | 0.70 |
| Return to dialysis and causes | 37 (13.5) | 36 (16.7) | 1 (1.7) | **0.02** |
| Rejection | 17/37 (46.0) | 17/36 (47.2) | 0/1 (0.0) | NA |
| Infection | 4/37 (10.8) | 4/36 (11.1) | 0/1 (0.0) |  |
| IFTA | 9/37 (24.3) | 9/36 (25.0) | 0/1 (0.0) |  |
| Vascular | 7/37 (18.9) | 6/36 (16.7) | 1/1 (100.0) |  |
| Causal nephropathy recurrence | 0/37 (0.0) | 0/36 (0.0) | 0/1 (0.0) |  |
| Death and causes | 22 (8.0) | 22 (10.2) | 0 (0.0) | **0.006** |
| Infection | 6/22 (27.3) | 6/22 (27.3) | − | NA |
| Cancer | 1/22 (4.5) | 1/22 (4.5) | − |  |
| Cardiovascular | 9/22 (40.9) | 9/22 (40.9) | − |  |
| Other | 6/22 (27.3) | 6/22 (27.3) | − |  |
| *Data are expressed as the number of patients (associated percentage) or median [25th; 75th percentiles]. cPRA, calculated panel reactive antibody; D0, day of transplantation; DSA, donor-specific antibody; G-CNI, group with immunosuppressive therapy maintenance; G-STOP, group with discontinued immunosuppressive therapy; HLA, human leucocyte antigen; IFTA, interstitial fibrosis and tubular atrophy; NA, not applicable.* | | | | |
