# Supplementary figures and images for: Impact of Calcineurin Inhibitor-Based Immunosuppression Maintenance During the Dialysis Period After Kidney Transplant Failure on the Next Kidney Graft Outcome: A Retrospective Multicenter Study With Propensity Score Analysis
Source: Transpl Int. 2023 Sep 15;36:11775. doi: 10.3389/ti.2023.11775 (PMC10548547; doi:10.3389/ti.2023.11775)

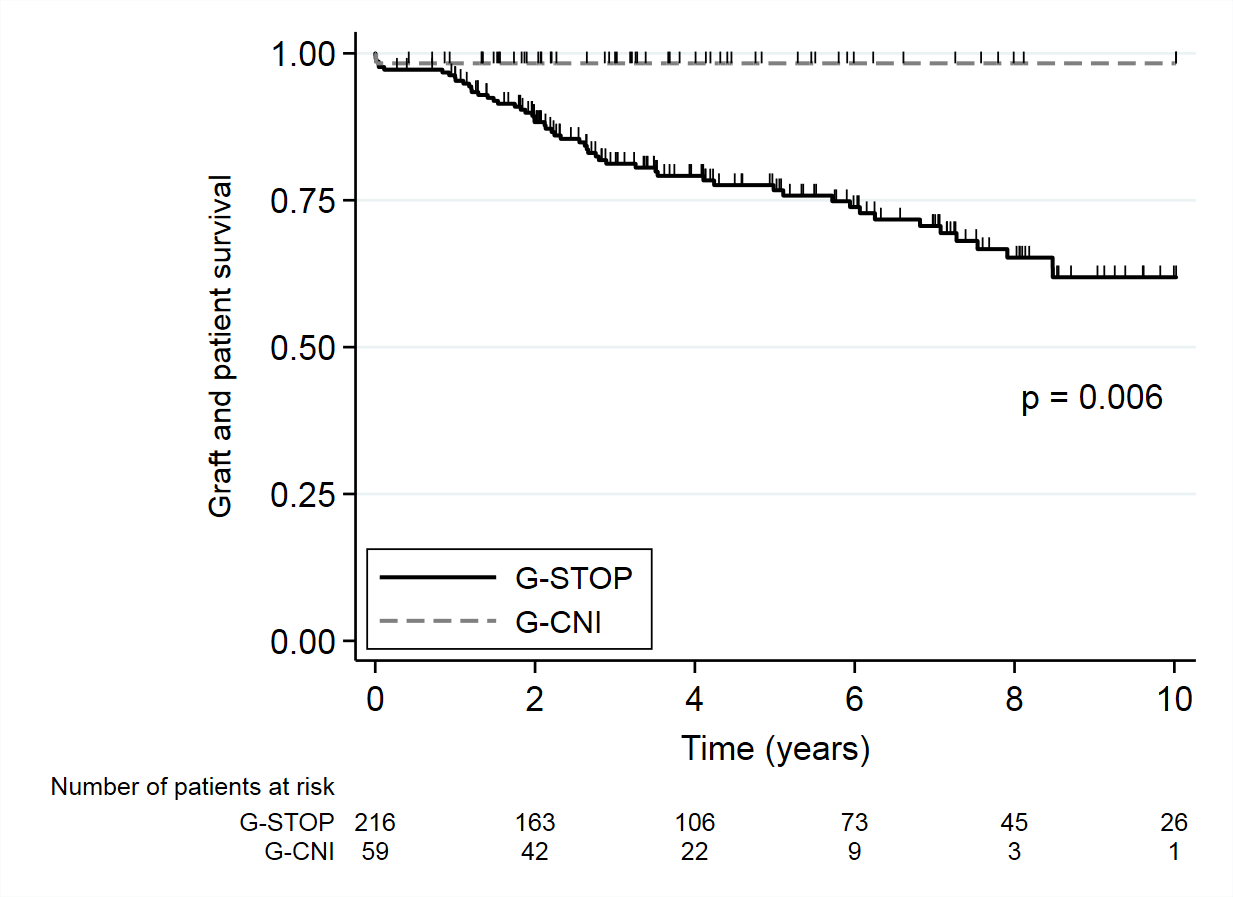

Supplement: Supplementary file 2 [file Image2.TIF]

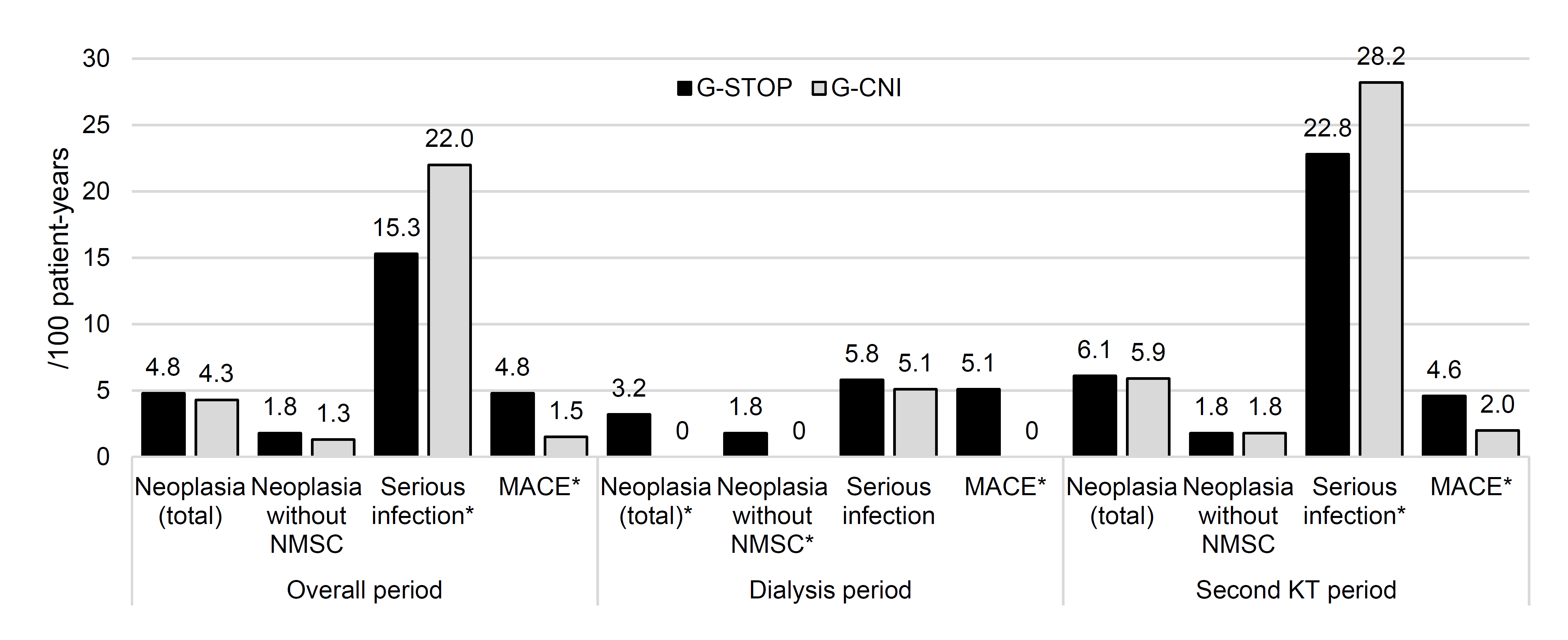

Supplement: Supplementary file 3 [file Image1.TIF]
